# Retinoic acid pathway activity in wilms tumors and characterization of biological responses *in vitro*

**DOI:** 10.1186/1476-4598-10-136

**Published:** 2011-11-08

**Authors:** Jenny Wegert, Sabrina Bausenwein, Susanne Kneitz, Sabine Roth, Norbert Graf, Eva Geissinger, Manfred Gessler

**Affiliations:** 1 Developmental Biochemistry, Biocenter, University of Wuerzburg, Wuerzburg, Germany; 2 Laboratory for Microarray Applications, University of Wuerzburg, Wuerzburg, Germany; 3 Institute of Pathology, University of Wuerzburg, Wuerzburg, Germany; 4 Division of Pediatric Oncology and Hematology, Saarland University Hospital, Homburg/Saar, Germany

**Keywords:** Wilms tumor, nephroblastoma, primary tumor cell culture, tumor model, retinoic acid

## Abstract

**Background:**

Wilms tumor (WT) is one of the most common malignancies in childhood. With current therapy protocols up to 90% of patients can be cured, but there is still a need to improve therapy for patients with aggressive WT and to reduce treatment intensity where possible. Prior data suggested a deregulation of the retinoic acid (RA) signaling pathway in high-risk WT, but its mode of action remained unclear.

**Results:**

The association of retinoid signaling and clinical parameters could be validated in a large independent tumor set, but its relevance in primary nephrectomy tumors from very young children may be different. Reduced RA pathway activity and MYCN overexpression were found in high risk tumors as opposed to tumors with low/intermediate risk, suggesting a beneficial impact of RA especially on advanced WT. To search for possible modes of action of retinoids as novel therapeutic options, primary tumor cell cultures were treated *in vitro *with all*-trans*-RA (ATRA), 9*cis-*RA, fenretinide and combinations of retinoids and a histone deacetylase (HDAC) inhibitor. Genes deregulated in high risk tumors showed opposite changes upon treatment suggesting a positive effect of retinoids. 6/7 primary cultures tested reduced proliferation, irrespective of prior RA signaling levels. The only variant culture was derived from mesoblastic nephroma, a distinct childhood kidney neoplasm. Retinoid/HDAC inhibitor combinations provided no synergistic effect. ATRA and 9*cis-*RA induced morphological changes suggestive of differentiation, while fenretinide induced apoptosis in several cultures tested. Microarray analysis of ATRA treated WT cells revealed differential expression of many genes involved in extracellular matrix formation and osteogenic, neuronal or muscle differentiation. The effects documented appear to be reversible upon drug withdrawal, however.

**Conclusions:**

Altered retinoic acid signaling has been validated especially in high risk Wilms tumors. *In vitro *testing of primary tumor cultures provided clear evidence of a potential utility of retinoids in Wilms tumor treatment based on the analysis of gene expression, proliferation, differentiation and apoptosis.

## Background

Wilms tumor (WT) - or nephroblastoma - is one of the most frequent solid tumors in childhood. This malignant kidney tumor affects about 1 of 10000 children. It arises from undifferentiated renal precursors and often presents with a triphasic histology consisting of blastemal, epithelial and stromal elements. Mutations of *CTNNB1*, *WT1 *or *WTX *were found in one third of WT, but in most cases the genetic etiology is still unclear [1]. Standard therapy according to the SIOP protocol consists of preoperative chemotherapy followed by tumor resection, or primary surgery for children under the age of six month. With current therapy overall survival rate can exceed 90% [2,3], but there is still a need for therapy improvement as prognosis of patients with high risk and relapsing WT is still poor.

In a previous study using a microarray strategy to detect new stratification markers for WT, the expression levels of several genes involved in the retinoic acid (RA) signaling pathway were found to be associated with disease progression [4]. These data suggested a contribution of RA signaling to tumor progression and RA treatment as an additional approach for therapy of WT. First hints on beneficial effects of RA were obtained when two primary WT cell cultures were treated with all-*trans *RA (ATRA) [5].

The vitamin A derivative ATRA is capable of inducing cell differentiation and inhibiting cell proliferation in various settings. It is already used in combination with chemotherapy in acute promyelocytic leukemia (APL). Retinoid therapy is also promising in pediatric malignancies, e.g. high risk neuroblastoma therapy using 13*cis*-RA [6]. While 13*cis*-RA is often administered in patients, it presumably acts as a pro-drug while ATRA represents the active form of RA [7]. Beside the classical retinoids ATRA, 13*cis*- or 9*cis*-RA the synthetic retinoid fenretinide (4HPR) is applied in cancer therapy. Whereas ATRA primarily induces differentiation, fenretinide may act via apoptosis/necrosis mechanisms [8].

Since WT originates from undifferentiated kidney precursor cells, ATRA-induced differentiation might be beneficial to improve patient's outcome. Furthermore, there is evidence that inhibitors of histone deacetylases may synergize with retinoic acid in inhibiting tumor growth, e.g. in childhood neuroblastoma [9,10]. Until today next to nothing is known about retinoids as therapeutic agents in WT, since only one case of 13*cis*-RA treatment of nephroblastomatosis, a WT precursor lesion, [11] and administration of fenretinide in one patient with WT [12] have been reported.

We have now validated prior microarray data in a much larger and independent set of 200 WT samples by realtime RT-PCR and we characterized the effects of RA treatment in an *in vitro *system of primary WT cultures. We used several different cell cultures established from fresh tumor material and treated them with classical and synthetic retinoids or a combination of retinoids and a histone deacetylase (HDAC)-inhibitor to evaluate potential synergy.

## Results

### Expression of RA pathway genes in WT

**Figure 1:**
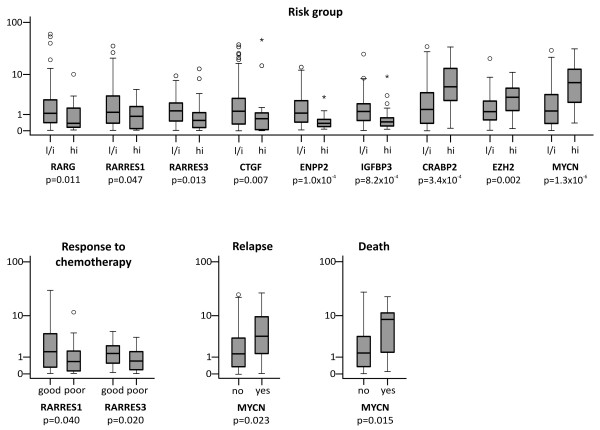
**Relative expression of RA pathway genes in Wilms tumor subclasses**. Box-plots represent 25% to 75% percentile and median of gene expression relative to mean of the left-hand subgroup in each pair. P-values are given for Mann-Whitney-U test comparing tumor subclasses for each gene tested. Risk group abbreviations: l/i, low/intermediate risk; hi, high risk

Prior data from microarray experiments [4] had pointed to deregulation of RA signaling pathway genes (RARB, RARG, RARRES1, RARRES3, ENPP2, CRABP2, CTGF, RAMP, MYCN, EZH2, PRAME and IGFBP3; Additional file 1, Table S1) in Wilms tumors. Here we sought to validate these findings in a much larger set of 200 WT samples (167 samples after chemotherapy and 33 after primary surgery). The following clinical criteria were evaluated: risk group (depending on histological subtype), response to chemotherapy, and occurrence of metastasis, relapse or death (Additional file 1, Table S2). The absolute numbers of metastasis, relapse or death cases comparable in the high risk vs. low/intermediate risk groups, but high risk tumors were of course less frequent (25 vs. 177). Comparison of WT after chemotherapy and primary resected specimens showed a higher expression of RA-inducible genes in post-chemotherapy WT. This could be in response to chemotherapy administration or due to differences in tumor biology of the two groups. We also detected a trend towards lower expression of these genes in post-chemotherapy specimens from younger vs. older patients. Omission of preoperative chemotherapy is only recommended for children under the age of 6 months, which have a much better prognosis and may represent a poorly separable, variant entity [13]. Tumors with primary surgery in our set are characterized by a much younger median age (4 vs. 35 months), but numbers are too small to generate statistically reliable data regarding the influence of age and/or therapy. Therefore, only tumor specimens with preoperative chemotherapy were included in subsequent statistical analysis. The Mann-Whitney-U test was used in an exploratory manner to compare expression levels of genes according to the criteria listed without adjustment of p-values to multiple testing. Detailed data on expression of all genes analyzed is summarized in Additional file 1, Table S3. The most prominent differences in gene expression were found when comparing low/intermediate vs. high risk tumors (Figure 1): RARG, RARRES1, RARRES3, CTGF, ENPP2 and IGFBP3 were downregulated, while CRABP2, EZH2 and MYCN were overexpressed in high risk WT. Furthermore, higher expression of MYCN was also seen in relapsing vs. non-relapsing and in fatal cases. Tumors with a poor response to chemotherapy, i.e. less than 50% reduction in volume during preoperative chemotherapy, showed lower expression of RARRES1 and RARRES3 compared to tumors with a strong decrease in tumor volume. Thus, we detect similar changes in RA pathway gene expression as described in Zirn et al. [4], especially with respect to risk classification and response to chemotherapy. Furthermore, we identify differential expression with reduced RA pathway activity in young age primary resected specimens.

### RA treatment of primary WT cultures

To gain additional insight into the action of RA on Wilms tumor cells and to test whether Wilms tumors could benefit from retinoid treatment, we used primary WT cell cultures as an *in vitro *system to study such effects. Seven primary WT cultures derived from five tumor samples (from 4 patients) were chosen for RA treatment (Additional file 1, Table S4). Three of them showed high baseline RA-signaling activity as measured by expression of RARA/B/G and RARRES1/2/3 (ws539A, ws539B and ws591) and they grew with a fibroblast-like mesenchymal phenotype. Four cultures exhibited low baseline RA-signaling activity (ws568li, ws568reA, ws568reB and ws592) with all ws568-derived cultures representing the mesenchymal phenotype, while ws592 was derived from a mesoblastic nephroma and consists of epithelial cells. The properties of all cell cultures have been described in detail elsewhere [14]. Baseline expression of RA pathway genes was generally lower in cultured cells compared to the original tumors, but the classification into high vs. low expressing cases remained unchanged.

To evaluate potentially divergent effects of clinically employed RA derivatives we tested three retinoids and one HDAC inhibitor. Each cell culture was treated with 10 μM ATRA, 9*cis*RA or 4HPR and the combination of 10 μM ATRA or 10 μM 4HPR together with 150 nM SAHA (HDAC inhibitor). These retinoid and SAHA concentrations have been used before in *in vitro *studies and can be reached in patients without severe side effects [5,6,10].

### Expression of RA pathway genes in treated WT cultures

**Figure 2:**
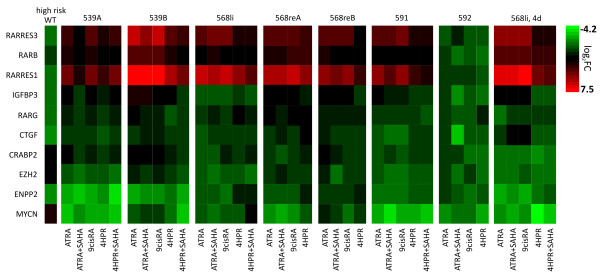
**Gene expression in RA treated WT cell cultures**. Primary WT cells were treated with the drugs indicated for 24 h or 4 days (568li, far right). Fold changes relative to untreated cells are depicted in the heat plot (log2 scaling). ATRA and 9*cis*RA caused similar changes of expression levels while 4HPR showed slight differences for some genes. There was no obvious effect of additional SAHA application.

Expression levels of genes differentially expressed in high vs. low/intermediate risk WT were measured by quantitative RT-PCR after 24 hours of treatment (this earlier time point should reduce the effects of secondary changes). Six of seven cultures showed concordant alterations in gene expression upon RA treatment (Figure 2, Additional file 1, Table S5): CRABP2, EZH2 and MYCN, which are over-expressed in high risk WT, were down-regulated by RA treatment in WT cultures. For CRABP2 changes were visible more clearly after 4 days of treatment. In all other cases tested there was no big difference between one and four days of treatment. RARB, RARRES1 and RARRES3 as well as IGFBP3 were up-regulated during RA administration, while RARG remained largely unchanged. For CTGF the direction of expression changes differed between the WT cultures used: it was slightly up-regulated in ws539 and ws568, but slightly down-regulated in ws591. Overall, the alterations in expression patterns of RA pathway genes were opposite to the changes seen in high risk vs. low/intermediate risk WT in six of seven cultures tested. Only the primary culture ws592 did not show comparable alterations of RA dependent genes and generally exhibited less than 2fold up-/down-regulation with any treatment.

There were no obvious differences in expression level changes between ATRA and 9*cis*RA treated cells. 4HPR elicited a similar response, but RARRES1/3, IGFBP3 and ENPP2 showed a reduced up-regulation. The HDAC inhibitor SAHA had no additional effect on expression levels beyond that seen with ATRA or 4HPR. Importantly, expression changes did not depend on basal RA-signaling activity, as alterations were rather similar in cell cultures with either low or high basal expression of RARA/B/G and RARRES1/2/3.

### Cell proliferation under RA treatment

**Figure 3:**
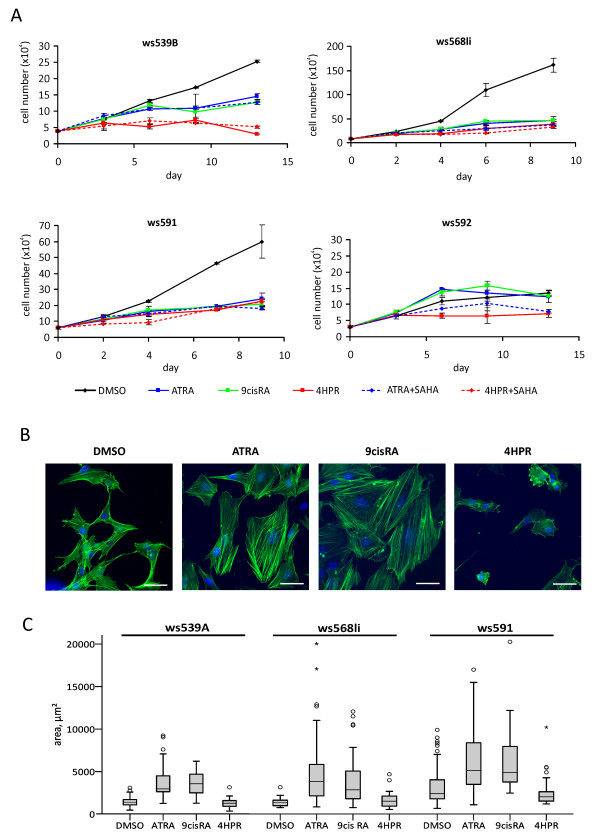
**Retinoids affect proliferation and cell morphology**. (A) Cell cultures ws539, ws568 and ws591 exhibit a decreased growth rate when treated with ATRA, 9*cis*RA or 4HPR. Cultures shown represent examples since different individual cultures derived from the same tumor sample/patient reacted in the same way. In culture ws592 no clear reduction of proliferation could be observed, except for 4HPR treatment. (B) Morphological changes under RA treatment as observed in WT cell culture ws568li. ATRA and 9*cis*RA induced a pronounced increase of cell size and formation of actin stress fibers. 4HPR rather led to cell shrinkage and detachment. Actin cytoskeleton is visualized in green (Phalloidin-FITC), nuclei are stained with Hoechst 33342 (blue), scale bar: 50 μm. (C) Numerical representation of the cell size changes upon treatment in cell lines ws539A, ws568li and ws591.

To analyze the impact of retinoids on proliferation of WT cells, cell numbers were determined under retinoic acid treatment for up to 14 days with all WT cultures used before (Figure 3A). All retinoids (ATRA, 9*cis*RA, 4HPR) strongly reduced proliferation in most of the cultures (ws539A, ws539B, ws568li, ws568reA, ws568reB and ws591). Growth rate of ws592 was not influenced, however, except for 4HPR, where a slight reduction was seen. For both cultures derived from tumor ws539 4HPR even exhibited a stronger effect than ATRA or 9*cis*RA, while there was no obvious difference in the ws568 and ws591 cultures. Again, the HDAC-inhibitor SAHA showed no additional effect on proliferation when given in combination with ATRA or 4HPR. Of note, independent cultures derived from one patient reacted in the same way (e.g. ws539A/ws539B and ws568li/ws568reA/ws568reB; Additional file 2, Figure S1).

In all experiments retinoid concentrations of 10 μM were used as these can be reached in patients as well. For WT culture ws568li treatment with 0.1 μM and 1 μM ATRA was tested in addition (Additional file 2, Figure S2). Both ATRA concentrations reduced the proliferation rate to the same extent as seen with 10 μM.

### RA treatment induces morphological changes

Primary cultures that respond to retinoids by alterations of gene expression and reduction of proliferation showed morphological changes after 4 days of treatment. Both classical retinoids (ATRA and 9*cis*RA) induced enlargement of cells with formation of strong actin fibers evident from phalloidin staining (Figure 3B). Cell size measurements (using an ellipsoid model) revealed an increase in cell size of 2.6-/2.5-fold (ws539A), 2.9-/3.5-fold (ws568li) and 2-/2.2-fold (ws591) with ATRA and 9*cis*RA, respectively (Figure 3C). 4HPR did not induce measurable changes, but in some cultures large numbers of cells died (ws539, ws568, see below). As before, ws592 cells neither exhibited morphological changes nor cell death under retinoid treatment.

An increase in cell size and flattening of the cell body is typical for senescent cells, but retinoid treated cells did not show typical multinucleation. To test whether retinoids induced a state of senescence, SA-β-Gal-staining was performed. Cultures treated for 4 days with ATRA or 9*cis*RA contained only few positive cells (less than 10%). When 4HPR was used, no senescent cells could be found (Additional file 2, Figure S3).

### Apoptosis induction by 4HPR

**Figure 4:**
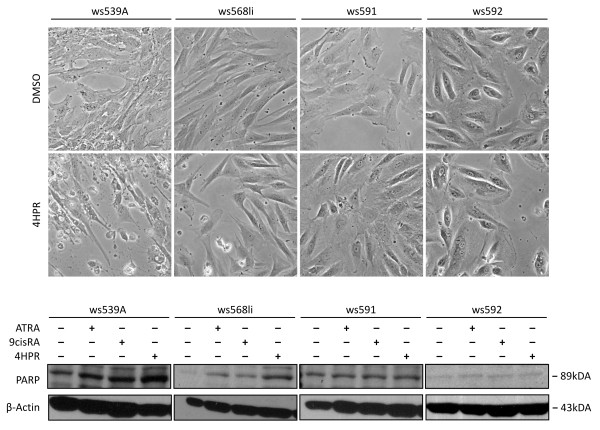
**4HPR induced apoptosis in WT cell cultures**. (A) 4HPR induced rounding and detachment of cells suggestive of cell death in cultures derived from tumors ws539 and ws568, while only few or no detached cell were found after 4d of treatment in cultures ws591 and ws592. (B) Increasing amounts of PARP cleavage as shown by Western blot analysis indicated apoptosis induction by 4HPR in WT cultures ws539 and ws568. ATRA and 9*cis*RA are less effective. For ws591 and ws592 no increase in apoptosis could be detected. β-actin was used as a loading control.

4HPR did not induce the morphological alterations seen for ATRA and 9*cis*RA, but many dead cells where found in 4HPR-treated ws539 cultures. In cultures ws568 and ws591 fewer cells were affected (Figure 4). Analysis of cleaved PARP as a late apoptosis marker revealed an increase in apoptosis in ws539 and ws568 cultures after 4 days of 4HPR treatment. With ATRA and 9*cis*RA only a slight induction of apoptosis could by detected in those cultures. In contrast, there was no obvious induction of apoptosis in cultures ws591 and ws592. IHC staining of ws539A cells for cleaved Caspase3 showed 80-100% apoptotic cells when treated with 4HPR or 4HPR+SAHA, while control ATRA or 9*cis*RA treated cells were negative for cleaved Caspase3 (Additional file 2, Figure S4).

### ATRA induces differentiation

To get insight into the response of primary WT cells to RA treatment we compared staining patterns for mesenchymal and epithelial markers used before in the characterization of the respective cell cultures. There were no striking or consistent changes after 4 days of treatment indicating that these cells did not fully change their phenotype upon treatment (based on CAM5.2, CD105, and CITED1; data not shown). To get a more global view of alterations induced by retinoids we compared gene expression profiles of control and ATRA treated (4 days) ws568li cells by microarray analysis (HG U133A Plus; Additional file 1, Table S6). 552 Genes were found to be up-regulated at least 2-fold (119 genes ≥ 4-fold) in ATRA-treated compared to control cells and 417 genes were down-regulated at least 2-fold (67 genes ≥ 4-fold).

**Figure 5:**
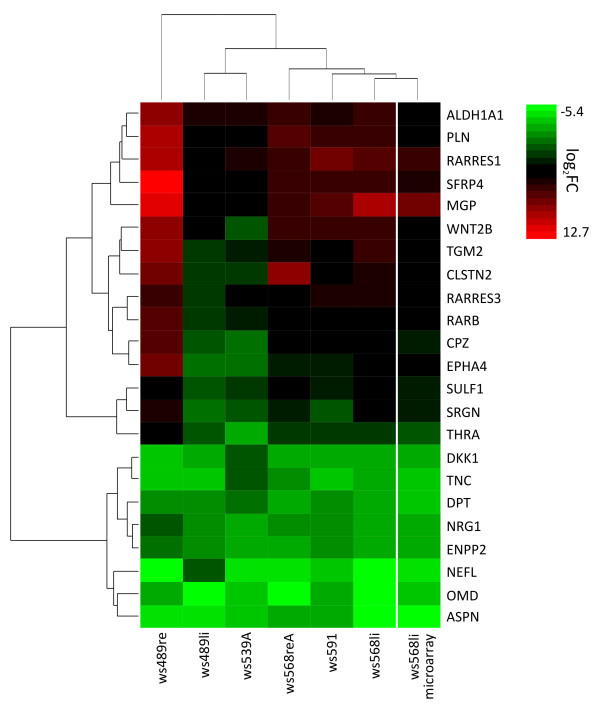
**Validation of microarray data by realtime RT-PCR**. Changes in gene expression after 4 days of ATRA administration found by microarray analysis in culture ws568li could be confirmed in different primary WT cells by realtime RT-PCR (heat map in log2 scaling).

To validate microarray data in other cell cultures quantitative RT-PCR was performed on control and ATRA-treated (4 days) samples of ws489li, ws489re, ws539A, ws568li, ws568reA and ws591 WT cultures. Genes from different functional groups were analyzed (Figure 5; Additional file 1, Table S7). All cultures tested showed up-regulation of RA metabolism/pathway genes as found in microarray analysis. For ws568li expression changes from the microarray data could be validated for all genes analyzed. Furthermore, all other cultures showed very similar regulation of gene expression upon ATRA treatment, albeit regulation is less prominent in cultures ws539A and ws489li, or more pronounced in ws489re.

Gene ontology analysis of differentially expressed genes (at least 2fold) identified several biological processes that appear to be strongly affected (Additional file 1, Table S8). Besides the expected changes affecting cell cycle and RA metabolism/signaling genes (enrichment score (ES) 19.4 and 3.1), these include genes playing a role in formation of the extra cellular matrix (ECM, ES 8.5). The expression of differentiation genes for bone/cartilage (ES 2.2), nervous (ES 3.3) and neural crest/mesenchymal lineages (ES 2.8) as well as for genes involved in angiogenesis (ES 6.2) was also altered more frequently. Among the genes with higher level alterations (> 4 ×) myogenic genes were enriched (ES 2.0). Nevertheless, these alterations in gene expression patterns do not point to a unidirectional differentiation, but rather to an induction of multiple differentiation pathways that may represent the plastic early embryonic state of these tumor cells.

### Long-term effects of retinoid treatment

**Figure 6:**
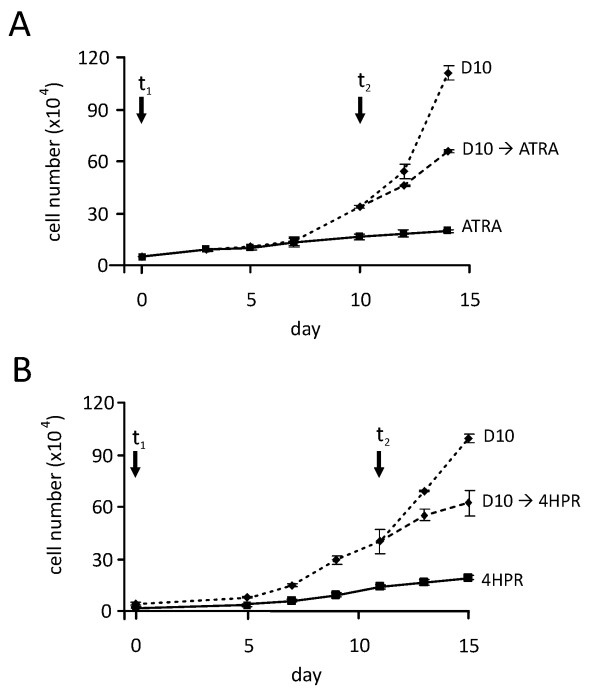
**Reversible effects of long-term retinoid treatment of WT culture ws568li**. (A) WT cells were continuously cultivated in the presence of 10 μM ATRA for 4 weeks. From time point t_1 _on cells were either kept in ATRA containing medium (ATRA) or cultivated in normal medium (D10). At time point t_2 _ATRA was given again (D10→ATRA) to one of the cultures. (B) WT cells were continuously cultivated in 10 μM 4HPR for 2 month. At time point t_1 _two cultures were switched to normal medium (D10), leading to an increase in proliferation after 5 days. If 4HPR was given again from time point t_2 _on cell growth was reduced again.

To study the effect of long-term ATRA treatment ws568li WT cells were kept in 10 μM ATRA containing medium for 4 weeks. Subsequent omission of ATRA led to an increase in proliferation within one week as compared to continuous ATRA treatment (Figure 6A). When ATRA was reapplied, growth rate was reduced again. Although 4HPR exhibited a strong repressive effect on cell proliferation and induced apoptosis, cells could still be kept under 10 μM 4HPR for longer periods of time. Similar to ATRA, removal of 4HPR reestablished proliferation and proliferation again declined upon renewed addition of 4HPR to the medium (Figure 6B). Both experiments suggest that neither ATRA nor 4HPR exhibit a persistent effect on WT cells and proliferation could increase again if retinoids were discontinued, even after long-term administration.

## Discussion

Although cure rate of WT is high with standard therapy, there is still a need for new therapeutic options, especially for the treatment of high risk and relapsing tumors. Additionally, a therapeutic strategy with fewer side effects as compared to classical chemotherapy would be desirable.

Our previous work provided first hints on deregulation of RA signaling in advanced WT, which may represent a starting point for new therapeutic approaches [4]. We therefore analyzed the expression of RA pathway genes in a larger, independent WT set to validate these findings. Again, deregulation of RA pathway genes in high risk vs. low/intermediate risk tumors was seen, albeit findings on relapsing tumors could not be confirmed at statistically significant levels. Others have described altered expression of RA pathway genes when comparing WT to fetal kidney [15]. In that study primary resected WT samples had been investigated, indicating that deregulation of RA signaling may be a general event in WT, independent of therapeutic strategy. Another study by Gupta and colleagues [16] revealed increased expression of CRABP2 in late stage Wilms tumors. There was evidence that this may be driven by elevated MYCN expression. In our samples we likewise found significantly elevated levels of MYCN in high risk, and relapsing tumors, but it remains to be tested if there is a direct relationship between MYCN levels and RA signaling activity e.g. in the different risk groups..

The comparatively smaller number of tumors in our study with primary surgery showed a lower expression of most RA pathway genes as compared to the larger cohort of post-chemotherapy samples. However, they are also characterized by a much earlier age at diagnosis (median age of 4 vs. 35 months). Most belong to a group of low risk tumors (age < 6 months, < 500 g, stage I, no preoperative chemotherapy) that are known to be curable by surgery alone as shown in NWTSG/COG [13]. Taking the data by Li et al. [15] into account, it thus appears that the differences in both, age and clinical subtype of our WT with primary surgery could be an important contributor to the divergent gene expression patterns observed between these two cohorts and levels of RA pathway activation may become more relevant only at slightly later ages. On the other hand, it is plausible that chemotherapy and concomitant local damage/inflammation may lead to an induction of RA signaling as part of a defense mechanism as seen in experimental glomerulonephritis [17]. Of note, exogenous RA had additional beneficial effects despite induction of the endogenous RA system in this case.

Since the available data point to the RA signaling pathway as an interesting target for WT therapy, we tested different retinoids in an *in vitro *model using primary WT cell cultures. Retinoid administration was able to inverse expression of genes found to be deregulated in high risk WT to a more favorable pattern characteristic for low/intermediate risk WT. Surprisingly, this effect was seen irrespective of the initial levels of expression of the corresponding genes in these cultures. Together with the strong growth suppression observed *in vitro *for all retinoids tested this clearly hints at a possible therapeutic utility of such a treatment. This is supported by the impressive clinical success of retinoid treatment in one case report of nephroblastomatosis, a WT precursor lesion, where a significant decline of kidney volumes was observed over a period of 1 year [11]. The comparatively poor response seen for ws592, a culture derived from mesoblastic nephroma, an early childhood tumor separate from WT, suggests that the effects observed with our cultures of classical WT may be rather specific and not due to general or unspecific effects of retinoids on cultured human cells and it may further set this tumor apart from classical WT.

Alteration of cell morphology under ATRA treatment points to an ATRA induced differentiation of WT cells, which is well known from other cell lines. WT specimens often contain different cell types like adipocytes, muscle cells, cartilage or bone structures or neuronal elements highlighting the differentiation potential of WT cells. We thus analyzed global gene expression changes in one of the treated WT cultures and we were able to validate these for selected genes in other cultures. Nevertheless, these patterns did not define a singular differentiation status or direction of ATRA treated WT cells, since a variety of genes involved in diverse differentiation processes were affected. This suggests that RA treatment may not induce terminal differentiation in treated WT cells, but partially supports multiple lineages.

The incomplete RA-induced differentiation is in line with results from long-term treatment, where neither ATRA nor 4HPR exhibited a persistent effect on WT cell morphology and where proliferation was again resumed if retinoids were discontinued. Thus, under *in vitro *conditions retinoids are not able to induce terminal differentiation, but they decrease cell proliferation. This would severely limit the usefulness of retinoid treatment since long-term treatment would lead to more significant side effects in young children. Similar results have been reported for rhabdomyosarcoma cells, where ATRA led to growth suppression and morphological changes, but these cells did not complete differentiation into multinucleated myotubes [18]. For neuroblastoma cell lines short-term ATRA treatment was sufficient to permanently reduce growth rate in some cases, but again there were cultures that increased proliferation upon ATRA removal [19]. An important caveat is that ATRA may act in a slightly different way in the *in vivo *situation, where tumor cells may encounter different endogenous stimuli as well as interactions with the ECM, immune cells or other neighboring cells that may ultimately fix differentiation and thereby contribute to tumor control.

In contrast to ATRA and 9*cis*RA, fenretinide did not lead to morphological changes indicative of differentiation, but rather induced apoptosis in most of the WT cells tested. Similar findings have been reported earlier for neuroblastoma cells: while ATRA drove differentiation and thus reduced overall cell proliferation, 4HPR induced growth arrest via induction of programmed cell death, without signs of differentiation [20]. As 4HPR can act independent of the typical RA signaling pathway through activation of ROS, lipid second messengers or mitochondrial pathways [21], it may represent an alternative approach, useful in ATRA resistant cases. Nevertheless, the similarity in gene expression patterns induced in treated cultures suggests that some overlap in signaling modes likely exists.

A further option for retinoid treatment could be the combination therapy with HDAC-inhibitors, as HDACs are part of the co-repressor complexes that inhibit expression of RA-target genes. Synergistic effects have already been described for APL cell lines where HDAC-inhibitors potentiate RA-induced differentiation and even restored RA-response in RA resistant cell lines [22]. The HDAC-inhibitor SAHA we used has been investigated before in neuroblastoma cell lines and an *in vivo *xenograft model [10], where combination therapy had a synergistic effect on differentiation and apoptosis and it improved host survival. However, in all our WT cell cultures SAHA exhibited no synergistic effect, neither in combination with ATRA nor 4HPR, suggesting that WTs may behave differently.

In summary, we provide novel insight into the response of WT cells to retinoic acid based treatment that suggests that retinoid administration may be an additional or alternative approach for therapy of Wilms tumors, esp. in those resistant to conventional therapy. Important caveats remain, however: *in vivo *models (e.g. xenografts in nude mice) are needed that better reflect the physiological situation in patients. Especially the reversibility of RA induced alterations *in vitro *must be critically assessed in the *in vivo *situation. Furthermore, the interplay of classical chemotherapy regimens based on cell damage with agents that promote differentiation and tumoristasis may prove difficult and again calls for improved modelling.

## Conclusions

We had initially identified altered retinoic acid signaling in different subgroups of Wilms tumors. These finding have now been extended and corroborated in a large set of 200 additional samples. Furthermore, we found evidence for age and stage/therapy-dependent expression of RA-pathway genes. We went on to evaluate the effects different retinoids on cultured primary Wilms tumor cells. We detected a strong decrease in proliferation that appears to be coupled to partial differentiation, especially in the case of classical retinoids. On the other hand, the synthetic derivative fenretinide seems to act primarily via induction of apoptosis. Nevertheless, all agents induced gene expression changes suggestive of partial differentiation in several directions. The cells remained in a rather plastic state as the antiproliferative effects of all retinoids were dependent on continuous presence of these agents. This is in line with results from other tumor entities and suggests that retinoids may supplement current therapeutic strategies, which is also evident from singular case reports in the literature.

## Materials and methods

### WT Samples and Clinical Data

Frozen tumor tissue and corresponding control samples (blood or normal kidney) were obtained from hospitals participating in the SIOP93-01/GPOH and SIOP2001/GPOH WT studies. Clinical data and reference pathology are from the central GPOH study registry. Patients categorized as relapse free had at least two years of follow-up, good response to chemotherapy was taken as a decrease in tumor volume of more than 50%.

### Isolation of DNA and RNA

Total RNA and DNA from tumor tissue and cell cultures were isolated using QIAGEN or Macherey-Nagel kits. Genomic DNA from kidney and blood samples were purified as described before [23].

### Realtime RT-PCR

2.5 μg of total RNA were used per cDNA synthesis reaction using the RevertAid First Strand cDNA synthesis kit (MBI Fermentas, St.Leon-Rot, Germany) with oligo dT primers. After cDNA synthesis water was added to a final volume of 200 μl. Realtime-PCR was conducted as described before with SybrGreen quantification [24]. Primers and PCR conditions used are listed in Additional file 1, Table S9. The housekeeping gene HPRT was used to normalize expression levels. All measurements were performed at least twice and mean values were calculated.

### Statistical analysis

Statistical analyses were performed with SPSS (Version 16.0). Mann-Whitney-U tests were used for comparison of expression level of genes analysed in the respective classes of metastasis, relapse, mortality, response to chemotherapy or histological subtype. The influence of RA treatment on WT cell size was tested in the same way.

### Cell culture and RA treatment

Primary WT cell cultures were maintained in Dulbecco's Modified Eagle Medium supplemented with 10% fetal calf serum and 1% penicillin/streptomycin (D10). Establishing and characterization of primary WT cell cultures has been described elsewhere [14]. Cells were treated with either 10 μM all-*trans *retinoic acid (ATRA, Sigma Aldrich), 10 μM 9-*cis *retinoic acid (9*cis*RA, Sigma Aldrich), 10 μM fenretinide (4-hydroxy(phenyl)retinamide, 4HPR, Sigma Aldrich), 10 μM ATRA + 0.15 μM of the HDAC-inhibitor suberoylanilide hydroxamic acid (N^1^-hydroxy-N^8^-phenyl-octanediamide, SAHA, Cayman Chemicals) or 10 μM 4HPR + 0.15 μM SAHA. Retinoid-containing medium was refreshed every second day. Untreated control cells received D10 with 1 μl/ml dimethyl sulfoxide that was used as solvent for retinoids.

### Determination of cell numbers

5 × 10^4 ^cells per well were seeded in 12well cell culture plates and allowed to adhere overnight. The following day (0 d) RA treatment was started. For each time point at least two samples were counted using a Neubauer chamber and mean values were calculated.

### Phalloidin staining

Cells were seeded on cover slips, incubated overnight and treated with retinoids as indicated for 4 days. Fixation was done with 2% paraformaldehyde in PBS (phosphate buffered saline) for 20 min at room temperature followed by washing and 10 min permeabilisation with PBS-T (PBS, 0.2% Tween). Actin filaments of cells were stained with 15 μg/ml of FITC conjugated Phalloidin (Sigma Aldrich) for 45 min and nuclei were counterstained with Hoechst 33342 (Invitrogen, 1:10, 000). Cover slips were mounted with Mowiol and cells were examined with an inverted microscope (Leica DMI 6000B). For cell size determination length and width of cells were measured using the microscope software (Leica Application Suite 1.7.0) and cell area was approximated using an ellipsoid model (A = 1/2*π*width*length).

### Senescence associated β-Gal-staining

Cells grown in 6-well cell dishes were washed with PBS (pH 7.2), fixed for 10 min with 0.5% glutaraldehyde and again washed with PBS (pH 7.2) + 1 mM MgCl_2_. Staining solution (1.2 ml) contained 1 mg/ml X-Gal (5-bromo-4-chloro-3-indolyl-beta-D-galacto-pyranoside), 0.12 mM K_3_Fe(CN)_6_, 0.12 mM K_4_Fe(CN)_6 _and 1 mM MgCl_2 _in PBS (pH 6.0). After 3 to 10 h of incubation at 37°C staining was stopped by washing with PBS + 1 mM MgCl_2 _(pH 7.2).

### Western blot analysis

5 × 10^6 ^cells were lysed in 50 μl RIPA buffer (150 mM NaCl, 100 mM Tris-HCl pH 7.4, 2% Nonidet P40, 1% sodium deoxycholate, 0.2% sodium dodecyl sulphate, 50 μg/μl phenylmethylsulfonyl fluoride and complete protease inhibitor cocktail (Roche)) for 45 min on ice, centrifuged for 30 min at 4°C and protein concentration of lysates was determined by Bradford assay (Biometra). Equal amounts of proteins were separated on 10% SDS-PAGE (sodium dodecyl sulfate polyacrylamide gel) and transferred to nitrocellulose membrane (PROTRAN, Schleicher&Schuell). Antibodies used for western blot analysis were cleaved PARP (1:1, 000; clone F21-852, BD Pharmingen), β-Actin (1:10, 000; clone C4, Santa Cruz Biotechnology) and mouse-IgG (1:5, 000; horseradish peroxidase conjugated, polyclonal AP124P, Chemicon). Detection was carried out using ECL chemiluminescent detection.

### Immunohistochemical analysis

Formalin fixed primary cells were used for immunohistochemical analysis. Preparation of cells blocks and staining procedure were described in detail in Wegert et al. [14]. Antibodies used were cleaved Caspase3 (rabbit monoclonal 5A1E, 1:100, Cell Signaling), CAM5.2 (1:5, BD Biosciences), CD105 (clone MEM-226, 1:200, Immunotools) and CITED1 (rabbit polyclonal, 1:100, Neo Markers)

### Microarray analysis

Total RNA of control and ATRA treated WT cells was used for microarray analysis on Human Genome U133 Plus 2.0 Gene Arrays (Affymetrix, Santa Clara, CA). Labeling and washing were performed according to the standard Affymetrix protocol. The arrays were scanned using a GeneChip^® ^Scanner 3000 (Affymetrix). Data analysis and quality control was done using different R packages from the Bioconductor project http://www.bioconductor.org. Probe sets were summarized using the RMA algorithm and resulting signal intensities were normalized by variance stabilization normalization (vsn) [25]. Functional clustering of differentially expressed genes was done with DAVID (Database for Annotation, Visualization and Integrated Discovery, [26]).

## Competing interests

The authors declare that they have no competing interests.

## Authors' contributions

JW and SB carried out most of the experiments, SK performed the microarray analysis and SR performed histopathological stainings. NG provided all clinical data and study organization, EG evaluated all immunostainings. MG designed and coordinated the study. JW, NG, EG and MG wrote the manuscript. All authors read and approved the final manuscript.

## Supplementary Material

Additional file 1 **Table S1: **RA pathway genes included in expression analysis.. **Table S2: **Statistics of expression of RA genes, all WT samples. **Table S3: **Statistics of expression of RA genes, WT with chemotherapy only. **Table S4: **Characteristics of primary WT cultures used. **Table S5: **Gene expression in RA treated WT cells (fold change compared to untreated cells). **Table S6: **Top 50 regulated genes of ATRA treated ws568li cells (microarray analysis). **Table S7: **Validation of microarray data by realtime RT-PCR (fold change). **Table S8: **Functional cluster analysis of ATRA regulated genes in WT cells. **Table S9: **Real-time RT-PCR primers and conditions. Click here for file

Additional file 2 **Figure S1**. All cell cultures derived from tumors of one patient reacted in the same way to RA treatment. Ws568reA and ws568reB showed reduction of growth rate under RA treatment like ws568li did. **Figure S2**. Low ATRA concentrations are sufficient to reduce cell growth. ws568li cells were treated with different ATRA concentrations, which all reduced cell proliferation to the same extent. **Figure S3**. SA-β-Gal staining of RA-treated ws568li cells. ATRA and 9cisRA induced only few senescent cells after 4d of administration while 4HPR treated cells showed no senescence. **Figure S4**. Caspase3 IHC staining of ws539A cells after 4d of RA treatment. Fenretinide and the combination of 4HPR and the HDAC inhibitor SAHA induced apoptosis in primary WT cells while ATRA, 9cisRA and ATRA combined with HDAC inhibitor did not. Click here for file
