# Supplementary material for: Retinoic acid pathway activity in wilms tumors and characterization of biological responses in vitro
Source: Mol Cancer. 2011 Nov 8;10:136. doi: 10.1186/1476-4598-10-136 (PMC3239322; doi:10.1186/1476-4598-10-136)
Supplement: Additional file 1 — Table S1: RA pathway genes included in expression analysis.. Table S2: Statistics of expression of RA genes, all WT samples. Table S3: Statistics of expression of RA genes, WT with chemotherapy only. Table S4: Characteristics of primary WT cultures used. Table S5: Gene expression in RA treated WT cells (fold change compared to untreated cells). Table S6: Top 50 regulated genes of ATRA treated ws568li cells (microarray analysis). Table S7: Validation of microarray data by realtime RT-PCR (fold change). Table S8: Functional cluster analysis of ATRA regulated genes in WT cells. Table S9: Real-time RT-PCR primers and conditions. [file 1476-4598-10-136-S1.PDF]

Additional file 1

Table S1: RA pathway genes included in expression analysis

Gene symbol	Gene name
CRABP2	Cellular retinoic acid binding protein 2
CTGF	Connective tissue growth factor
ENPP2	Ectonucleotide pyrophosphatase/ phosphodiesterase 2
EZH2	Enhancer of zeste homolog 2
IGFBP3	Insulin-like growth factor binding protein 3
MYCN	v-myc myelocytomatosis viral related oncogene, neuroblastoma derived
PRAME	Preferentially expressed antigen in melanoma
RAMP	Receptor activity-modifying protein
RARB	Retinoic acid receptor, beta
RARG	Retinoic acid receptor, gamma
RARRES1	Retinoic acid receptor responder (tazarotene induced) 1
RARRES3	Retinoic acid receptor responder (tazarotene induced) 3

Table S2: Statistics of expression of RA genes, all WT samples

Gene	Response to chemotherapy good - poor	Risk low/intermediate - high	Relapse no – yes	Metastasis no – yes	Death no – yes	chemotherapy – primary surgery
**CRABP2**	25.00 – 3.11<br>24.40 – 2.16<br>p=0.378<br>N=54/35	24.94 – 3.14<br>23.10 – 2.23<br>p=7.57E-4<br>N=177/25	24.71 – 3.12<br>24.41 – 2.96<br>p=0.764<br>N=181/14	24.68 – 3.08<br>24.76 – 3.60<br>p=0.869<br>N=180/14	24.74 – 3.12<br>23.55 – 2.78<br>p=0.215<br>N=187/9	24.86 – 3.16<br>23.91 – 2.64<br>p=0.051<br>N=170/31
**CTGF**	29.14 – 2.82<br>30.11 – 2.69<br>p=0.076<br>N=54/35	29.40 – 2.69<br>30.92 – 3.39<br>p=0.019<br>N=177/25	29.70 – 2.77<br>28.89 – 3.41<br>p=0.478<br>N=181/14	29.59 – 2.80<br>30.14 – 3.16<br>p=0.399<br>N=180/14	29.66 – 2.88<br>29.13 – 1.92<br>p=0.791<br>N=187/9	29.29 – 2.78<br>31.27 – 2.05<br>p=2.23E-5<br>N=170/31
**ENPP2**	26.27 – 1.93<br>26.29 – 1.89<br>p=0.737<br>N=54/35	26.24 – 1.82<br>27.54 – 1.43<br>p=5.23E-4<br>N=177/25	26.39 – 1.86<br>26.91 – 1.67<br>p=0.253<br>N=181/14	26.37 – 1.86<br>26.91 – 1.73<br>p=0.273<br>N=180/14	26.41 – 1.87<br>26.67 – 1.51<br>p=0.650<br>N=187/9	26.19 – 1.77<br>27.55 – 1.62<br>p=1.26E-4<br>N=170/31
**EZH2**	33.74 – 1.96<br>33.34 – 2.09<br>p=0.253<br>N=52/34	33.65 – 2.01<br>32.57 – 1.65<br>p=0.007<br>N=171/24	33.54 – 2.02<br>33.46 – 2.00<br>p=0.931<br>N=174/14	33.52 – 1.97<br>33.63 – 2.65<br>p=0.926<br>N=174/13	33.54 – 1.98<br>32.92 – 2.64<br>p=0.355<br>N=180/9	33.64 – 2.03<br>32.96 – 1.81<br>p=0.118<br>N=163/31
**IGFBP3**	23.31 – 1.97<br>23.20 – 1.77<br>p=0.983<br>N=54/35	23.25 – 1.82<br>24.26 – 1.68<br>p=0.002<br>N=177/25	23.40 – 1.87<br>23.49 – 1.66<br>p=0.658<br>N=181/14	23.34 – 1.81<br>24.39 – 2.26<br>p=0.098<br>N=180/14	23.40 – 1.88<br>23.81 – 1.28<br>p=0.353<br>N=187/9	23.22 – 1.83<br>24.25 – 1.57<br>p=0.001<br>N=170/31
**MYCN**	30.04 – 2.40<br>29.79 – 2.17<br>p=0.723<br>N=53/35	30.18 – 2.60<br>27.69 – 1.62<br>p=2.18E-6<br>N=176/25	30.01 – 2.64<br>28.63 – 2.64<br>p=0.037<br>N=180/14	29.95 – 2.57<br>29.57 – 3.46<br>p=0.496<br>N=179/14	30.02 – 2.62<br>28.08 – 2.55<br>p=0.020<br>N=186/9	29.93 – 2.58<br>29.76 – 2.90<br>p=0.531<br>N=169/31
**PRAME**	32.11 – 3.49<br>31.71 – 3.13<br>p=0.846<br>N=52/34	32.08 – 3.23<br>31.27 - 3.38<br>p=0.156<br>N=172/24	32.05 – 3.25<br>31.63 – 3.57<br>p=0.521<br>N=175/14	32.01 – 3.17<br>32.30 – 4.52<br>p=0.959<br>N=174/14	32.03 – 3.21<br>31.29 – 4.61<br>p=0.317<br>N=181/9	31.75 – 3.25<br>33,23 – 3.02<br>p=0.008<br>N=164/31
**RAMP**	27.62 – 1.60<br>27.24 – 1.59<br>p=0.575<br>N=54/34	27.70 – 1.91<br>27.68 – 1.25<br>p=0.734<br>N=174/24	27.68 – 1.86<br>27.88 – 1.63<br>p=0.612<br>N=177/14	27.69 – 1.84<br>27.59 – 1.96<br>p=0.587<br>N=176/14	27.69 – 1.86<br>27.30 – 1.51<br>p=0.487<br>N=183/9	27.68 – 1.88<br>27.76 – 1.72<br>p=0.856<br>N=166/31
**RARB**	26.14 – 1.51<br>26.40 – 1.18<br>p=0.183<br>N=54/35	26.30 – 1.45<br>26.32 – 1.15<br>p=0.586<br>N=177/25	26.27 – 1.41<br>26.83 – 1.56<br>p=0.179<br>N=181/14	26.24 – 1.37<br>26.98 – 1.82<br>p=0.144<br>N=180/14	26.20 – 1.43<br>26.14 – 1.05<br>p=0.814<br>N=187/9	26.24 – 1.39<br>26.66 – 1.44<br>p=0.170<br>N=170/31
**RARG**	31.07 – 2.15<br>31.02 – 2.71<br>p=0.963<br>N=53/35	30.92 – 2.31<br>31.97 – 2.06<br>p=0.017<br>N=176/25	31.01 – 2.30<br>31.56 – 2.35<br>p=0.375<br>N=180/14	30.96 – 2.20<br>32.21 – 3.29<br>p=0.269<br>N=179/14	31.03 – 2.33<br>31.40 – 2.07<br>p=0.635<br>N=186/9	30.89 – 2.27<br>31.27 – 2.05<br>p=0.023<br>N=169/31
**RARRES1**	27.90 – 2.72<br>29.14 – 2.70<br>p=0.040<br>N=54/35	28.61 – 2.70<br>29.62 – 2.78<br>p=0.177<br>N=177/25	28.76 – 2.79<br>28.49 – 2.40<br>p=0.651<br>N=181/14	28.76 – 2.79<br>28.42 – 2.49<br>p=0.662<br>N=180/14	28.74 – 2.81<br>28.22 – 1.56<br>p=0.976<br>N=187/9	28.35 – 2.55<br>30.62 – 2.77<br>p=6.36E-6<br>N=170/31
**RARRES3**	30.54 – 1.61<br>31.54 – 1.99<br>p=0.020<br>N=54/35	30.85 – 2.01<br>31.94 – 2.05<br>p=0.031<br>N=177/25	30.96 – 2.06<br>30.89 – 2.21<br>p=0.829<br>N=181/14	30.95 – 2.06<br>30.94 – 2.24<br>p=0.949<br>N=180/14	30.98 – 2.08<br>30.46 – 1.73<br>p=0.654<br>N=187/9	30.71 – 1.82<br>32.10 – 2.71<br>p=0.003<br>N=170/31

Numbers represent qRT-PCR data: Mean C_t-value – Standard deviation (first subgroup); Mean C_t-value – Standard deviation (second subgroup); p: p-value (Mann-Whitney-U test); N: number of cases each subgroup

Table S3: Statistics of expression of RA genes, WT with chemotherapy only

Gene	Response to chemotherapy good - poor	Risk low/intermediate - high	Relapse no – yes	Metastasis no – yes	Death no – yes
**CRABP2**	24.98-3.13<br>24.40-2.16<br>p=0.378<br>N=54/35	25.14 -3.21<br>23.10-2.28<br>p=3.73E-4<br>N=143/24	24.93-3.23<br>24.42-2.96<br>p=0.592<br>N=148/14	24.94-3.22<br>24.23-3.13<br>p=0.390<br>N=148/13	24.95-3.22<br>23.55-2.78<br>p=0.165<br>N=153/9
**CTGF**	29.13-2.85<br>30.12-2.69<br>p=0.076<br>N=54/35	29.03 -2.63<br>30.72-3.31<br>p=0.007<br>N=143/24	29,33-2,75<br>28,89-3,41<br>p=0.837<br>N=148/14	29.23-2.80<br>29.77-2.96<br>p=0.3092<br>N=148/13	29.32-2.86<br>29.13-1.92<br>p=0.792<br>N=153/9
**ENPP2**	26.27-1.94<br>26.29-1.89<br>p=0.737<br>N=54/35	25.97-1.74<br>27.41-1.30<br>p=1.02E-4<br>N=143/24	26.12-1.78<br>26.91-1.68<br>p=0.098<br>N=148/14	26.1-1.78<br>26.80-1.76<br>p=0.193<br>N=148/13	26.16-1.81<br>26.67-1.51<br>p=0.378<br>N=153/9
**EZH2**	33.70-1.95<br>33.34-2.09<br>p=0.253<br>N=52/34	33.81-2.03<br>32.55-1.68<br>p=0.002<br>N=137/23	33.69-2.05<br>33.46-2,00<br>p=0.881<br>N=141/14	33.69-2,00<br>33.38-2.62<br>p=0.616<br>N=142/12	33.69-2.02<br>32.92-2.64<br>p=0.277<br>N=146/9
**IGFBP3**	23.24-1.92<br>23.20-1.77<br>p=0.983<br>N=54/35	23.05-1.81<br>24.17.66<br>p=8.19E-4<br>N=143/24	23.20-1.87<br>23.49-1.66<br>p=0.368<br>N=148/14	23.15-1.82<br>24.13-2.12<br>p=0.102<br>N=148/13	23.22-1.88<br>23.81-1.28<br>p=0.182<br>N=153/9
**MYCN**	30.00-2.40<br>29.79-2.17<br>p=0.723<br>N=53/35	30.28-2.53<br>27.73-1.64<br>p=1.27E-6<br>N=142/24	30.08-2.57<br>28.63-2.64<br>p=0.023<br>N=147/14	30.03-2.52<br>29.14-3.21<br>p=0.215<br>N=147/13	30.10-2.56<br>28.08-2.55<br>p=0.015<br>N=152/9
**PRAME**	32.06-3.50<br>31.71-3.13<br>p=0.846<br>N=52/34	31.84-3.23<br>31.24-3.45<br>p=0.310<br>N=138/23	31.80-3.25<br>31.63-3.58<br>p=0.754<br>N=142/14	31.79-3.17<br>31.89-4.42<br>p=0.735<br>N=142/13	31.81-3.20<br>31.29-4.61<br>p=0.395<br>N=147/9
**RAMP**	27.57-1.57<br>27.24-1.57<br>p=0.575<br>N=54/34	27.70-1.97<br>27.49-1.27<br>p=0.618<br>N=140/23	27.69-1.93<br>27.88-1.63<br>p=0.620<br>N=144/14	27.74-1.93<br>27.22-1.42<br>p=0.272<br>N=144/13	27.70-1.93<br>27.30-1.51<br>p=0.481<br>N=149/9
**RARB**	26.08-1.46<br>26.40-1.18<br>p=0.183<br>N=54/35	26.24-1.44<br>26.26-1.14<br>p=0.595<br>N=143/24	26.19-1.39<br>26.83-1.56<br>p=0.141<br>N=148/14	26.17-1.37<br>26.77-1.70<br>p=0.250<br>N=148/13	26.24-1.41<br>26.14-1.05<br>p=0.861<br>N=153/9
**RARG**	31.00-2.09<br>31.02-2.71<br>p=0.963<br>N=53/35	30.71-2.26<br>31.95-2.11<br>p=0.011<br>N=142/24	30.83-2.28<br>31.56-2.35<br>p=0.268<br>N=147/14	30.83-2.27<br>31.61-2.51<br>p=0.375<br>N=147/13	30.87-2.31<br>31.40-2.07<br>p=0.505<br>N=152/9
**RARRES1**	27.93-2.74<br>29.14-2.70<br>p=0.0396<br>N=54/35	28.18-2.51<br>29.43-2.66<br>p=0.047<br>N=143/24	28.34-2.60<br>28.49-2.40<br>p=0.941<br>N=148/14	28.38-2.62<br>28.02-2.06<br>p=0.717<br>N=148/13	28.35-2.64<br>28.44-1.56<br>p=0.604<br>N=153/9
**RARRES3**	30.54-1.62<br>31.54-1.99<br>p=0.020<br>N=54/35	30.58-1.73<br>31,54-2.21<br>p=0.013<br>N=143/24	30.69-1,80<br>30.90-2.21<br>p=0.535<br>N=148/14	30.72-1.84<br>30.54-1.73<br>p=0.867<br>N=148/13	30.73-1,86<br>30.46-1.73<br>p=0.892<br>N=153/9

Numbers represent qRT-PCR data: Mean C_t-value – Standard deviation (first subgroup); Mean C_t-value – Standard deviation (second subgroup); p: p-value (Mann-Whitney-U test); N: number of cases each subgroup

Table S4: Characteristics of primary WT cultures used

Case (ws)	Age (m)	sex[a]	Chemo-therapy	Laterality	Histology	Mutations, LOH [c]	Cell culture	Growth [d]	Max. passages	Morphology	IHC
**489**	9	m	yes	left	stromal	CTNNB1 ΔS45 (het), WT1 R362X (hom), LOH 11p	ws489li-1	fast	13	fibroblast-like	CD105^{+} Cam5.2^{-}
							ws489li-2	fast	12	fibroblast-like	
				right	triphasic	CTNNB1 T41A (het), WT1 R362X (hom), LOH 11p	ws489re	fast	13	fibroblast-like	CD105^{+} Cam5.2^{-}
**539**	53	f	yes	left	stromal	LOH 7p, 11p, Δ WTX (het)	ws539A	fast	30	fibroblast-like	CD105^{+} Cam5.2^{-}
							ws539B	fast	28	fibroblast-like	
**568**	9	f	yes	left	stromal	CTNNB1 S45F (het), WT1 Q184X (het), LOH 11p	ws568li	fast	34	fibroblast-like	CD105^{+} Cam5.2^{-}
				right	triphasic	CTNNB1 ΔS45 (het), WT1 Q184X (het), LOH 11p	ws568reA	fast	30	fibroblast-like	CD105^{+} Cam5.2^{-}
							ws568reB	fast	28	fibroblast-like	
**591**	62	m	yes	right	stromal	Δ WTX	ws591	fast	17	fibroblast-like	CD105^{+} Cam5.2^{-}
**592**	2	m	no	right	CMN[b]	LOH 11p	ws592	slow	8	round (epith.)	CD105^{-} Cam5.2^{+}

a: f: female, m: male

b: CMN: congenital mesoblastic nephroma

c: hom: homozygous, het: heterozygous

d: fast: subculturing at a ratio of 1:3 at least once a weak, slow: subculturing less frequent

Table S5: Gene expression in RA treated WT cells (fold change compared to untreated cells)

	ws539A					ws539B					ws568li					ws568reA				ws568reB				ws591					ws592				ws568li, 4d				
	ATRA	ATRA+SAHA	9cis RA	4HPR	4HPR+SAHA	ATRA	ATRA+SAHA	9cis RA	4HPR	4HPR+SAHA	ATRA	ATRA+SAHA	9cis RA	4HPR	4HPR+SAHA	ATRA	ATRA+SAHA	9cis RA	4HPR	ATRA	ATRA+SAHA	9cis RA	4HPR	ATRA	ATRA+SAHA	9cis RA	4HPR	4HPR+SAHA	ATRA	ATRA+SAHA	9cis	4HPR	ATRA	ATRA+SAHA	9cis RA	4HPR	4HPR+SAHA
RARRES3	10,56	3,52	10,85	5,56	7,81	54,38	33,94	62,90	11,92	7,94	14,27	17,57	16,85	6,36	6,89	15,40	11,51	15,56	9,99	15,03	13,22	11,43	2,75	13,59	11,92	16,97	4,74	5,88	0,84	1,51	0,66	0,63	34,42	25,37	27,86	5,19	4,82
RARB	5,28	2,20	5,37	2,68	4,55	13,45	12,68	14,93	5,15	3,72	4,23	4,72	4,56	3,45	4,23	6,28	6,54	7,57	8,34	5,33	2,70	4,00	2,97	3,80	2,77	2,57	3,15	2,72	1,17	0,50	0,74	0,49	11,35	15,19	15,03	8,54	7,14
RARRES1	23,67	4,74	22,09	7,59	16,97	135,77	130,69	181,02	47,67	20,32	73,77	36,00	59,71	24,93	12,60	51,09	40,79	57,68	26,91	31,02	17,39	28,64	7,94	26,45	14,52	27,57	8,66	5,96	1,14	1,13	1,01	0,75	119,02	91,77	131,14	17,33	10,82
IGFBP3	3,56	1,29	2,91	2,02	3,23	4,87	5,21	3,52	2,11	0,96	0,68	0,87	0,80	0,97	0,68	3,97	3,94	2,74	1,91	3,89	2,75	2,27	1,27	2,58	1,60	1,89	1,25	1,39	0,96	0,28	0,73	0,48	2,38	4,01	3,42	0,82	0,76
RARG	1,45	1,21	1,57	1,23	1,62	2,25	1,89	1,91	0,91	1,02	1,30	1,54	1,38	2,26	1,82	2,43	1,68	2,45	2,23	1,90	1,69	1,62	1,65	1,29	1,13	1,16	1,14	0,91	0,98	0,81	0,80	1,41	0,57	1,38	1,15	1,21	1,18
CTGF	1,03	0,98	1,26	0,81	0,95	1,11	1,67	1,48	0,97	1,00	0,74	1,29	1,07	0,97	1,13	1,24	1,66	1,05	2,69	1,55	1,24	1,10	1,30	0,65	0,53	0,45	1,34	1,33	0,94	0,17	0,71	0,68	1,23	2,70	2,26	0,64	0,72
CRABP2	2,52	0,97	2,07	1,05	1,98	4,39	3,31	3,96	1,83	1,31	0,91	0,93	1,40	1,21	1,66	0,97	0,92	1,15	1,45	2,58	1,62	1,31	1,35	0,68	0,95	0,85	0,95	1,35	1,02	1,34	0,50	0,52	0,46	0,54	0,55	0,36	0,60
EZH2	0,80	0,50	0,85	0,69	0,50	1,51	1,30	1,51	1,21	0,73	0,65	0,51	0,65	0,64	0,49	1,10	1,44	1,35	1,14	1,48	0,60	1,04	1,30	0,85	0,44	0,57	0,84	0,72	0,97	0,47	1,07	0,54	0,46	0,52	0,74	0,61	0,55
ENPP2	0,20	0,16	0,26	0,32	0,12	0,27	0,29	0,37	0,53	0,25	0,63	0,69	0,58	1,40	1,50	0,64	0,71	0,64	1,88	1,33	1,04	1,15	1,45	0,47	0,52	0,45	0,88	0,92	0,94	0,77	0,50	0,68	0,24	0,33	0,27	0,55	0,71
NMYC	0,18	0,39	0,27	0,28	0,17	0,68	1,00	1,06	0,42	0,17	0,67	0,47	0,90	0,91	2,54	0,30	0,22	0,41	0,81	1,99	1,14	0,49	0,98	0,18	0,11	0,19	0,24	0,14	0,38	0,51	0,45	0,19	0,36	0,22	0,29	0,06	0,17

Table S6: Top 50 regulated genes of ATRA treated ws568li cells (microarray analysis)

Gene name	Gene symbol	Fold change
complement factor I	CFI	178.1389
complement factor I	CFI	123.4127
matrix Gla protein	MGP	116.3096
cytochrome P450, family 26, subfamily B, polypeptide 1	CYP26B1	102.2871
capping protein (actin filament) muscle Z-line, alpha 1	CAPZA1	65.2998
retinoic acid receptor responder (tazarotene induced) 1	RARRES1	63.3247
matrix Gla protein	MGP	48.4251
retinoic acid receptor responder (tazarotene induced) 1	RARRES1	48.3828
asporin	ASPN	0.0231
leucine rich repeat containing 15	LRRC15	0.0245
retinoic acid receptor responder (tazarotene induced) 1	RARRES1	38.6526
natriuretic peptide precursor B	NPPB	36.1269
suppression of tumorigenicity 7 like	ST7L	36.1050
secreted frizzled-related protein 4	SFRP4	28.4684
clusterin-like 1 (retinal)	CLUL1	26.7697
hyaluronan synthase 2	HAS2	0.0401
asporin	ASPN	0.0447
WD repeat domain 86	WDR86	22.3004
wingless-type MMTV integration site family, member 2B	WNT2B	20.9623
dehydrogenase/reductase (SDR family) member 3	DHRS3	20.1865
thrombospondin, type I, domain containing 7A	THSD7A	18.9862
dehydrogenase/reductase (SDR family) member 3	DHRS3	18.7228
secreted frizzled-related protein 4	SFRP4	17.6783
collagen, type XXI, alpha 1	COL21A1	0.0574
tumor-associated calcium signal transducer 2	TACSTD2	17.2989
hyaluronan synthase 2	HAS2	0.0594
neurofilament, light polypeptide	NEFL	0.0602
transglutaminase 2 (C polypeptide, protein-glutamine-gamma-glutamyltransferase)	TGM2	16.4632
C-type lectin domain family 3, member B	CLEC3B	0.0610
thrombospondin, type I, domain containing 7A	THSD7A	16.2578
thrombospondin, type I, domain containing 7A	THSD7A	15.6809
Kruppel-like factor 4 (gut)	KLF4	0.0649
endoplasmic reticulum protein 27	ERP27	15.1880
thrombospondin, type I, domain containing 7A	THSD7A	15.1632
aldehyde dehydrogenase 1 family, member A1	ALDH1A1	15.1277
latexin	LXN	14.7063
EGF-containing fibulin-like extracellular matrix protein 1	EFEMP1	14.0814
glutathione peroxidase 3 (plasma)	GPX3	13.7874
solute carrier family 14 (urea transporter), member 1 (Kidd blood group)	SLC14A1	0.0732
EPH receptor A4	EPHA4	13.6486
regulator of G-protein signaling 2, 24kDa	RGS2	13.0383
phospholamban	PLN	12.8144
EPH receptor A4	EPHA4	12.7345
glypican 6	GPC6	11.6986
POM121 membrane glycoprotein-like 9 (rat) pseudogene	POM121L9P	11.6564
neurofilament, light polypeptide	NEFL	0.0866
cornichon homolog 3 (Drosophila)	CNIH3	0.0896
calsyntenin 2	CLSTN2	11.1101
keratin 18	KRT18	10.9777

Table S7: Validation of microarray data by realtime RT-PCR (fold change)

Gene symbol	Gene name	Fold change						
		**ws489re**	**ws489li**	**ws539A1-2**	**ws568reA**	**ws591**	**ws568li**	**ws568li array**
**ALDH1A1**	Aldehyde dehydrogenase 1 family, member A1	517.35	23.34	33.36	75.06	32.00	56.69	15.13
**PLN**	Phospholamban	741.86	8.51	9.29	123.21	49.69	58.89	12.81
**RARRES1**	Retinoic acid receptor responder (tazarotene induced) 1	652.58	13.55	23.67	58.89	190.68	119.02	63.32
**SFRP4**	Secreted frizzled-related protein 4	6746.86	12.60	17.57	64.45	70.28	55.14	28.47
**MGP**	Matrix Gla protein	2503.97	8.66	11.96	46.69	114.17	823.14	178.14
**WNT2B**	Wingless-type MMTV integration site family, member 2B	386.68	11.71	1.70	65.57	46.21	65.57	20.96
**TGM2**	Transglutaminase 2	293.05	2.18	4.82	43.11	7.29	70.52	16.46
**CLSTN2**	Calsyntenin 2	266.87	2.69	2.08	455.09	20.89	31.23	11.11
**RARRES3**	Retinoic acid receptor responder (tazarotene induced) 3	60.34	2.97	10.56	10.74	39.26	34.42	8.69
**RARB**	Retinoic acid receptor, beta	104.69	2.23	5.28	19.36	17.27	11.35	9.69
**CPZ**	Carboxypeptidase Z	146.02	1.19	0.71	12.04	10.67	15.14	5.26
**EPHA4**	EPH receptor A4	203.66	0.85	0.62	6.19	4.59	22.78	13.65
**SULF1**	Sulfatase 1	7.81	1.61	2.07	7.84	4.26	10.67	6.18
**SRGN**	Serglycin	24.00	0.78	1.66	4.56	1.29	9.42	5.96
**THRA**	Thyroid hormone receptor, alpha	12.00	1.00	0.22	2.06	2.59	2.32	1.43
**DKK1**	Dickkopf homolog 1	0.15	0.25	1.19	0.28	0.15	0.23	0.23
**TNC**	Tenascin C	0.12	0.14	1.27	0.45	0.12	0.25	0.11
**DPT**	Dermatopontin	0.39	0.44	0.68	0.20	0.40	0.17	0.14
**NRG1**	Neuregulin 1	1.03	0.51	0.28	0.35	0.29	0.25	0.20
**ENPP2**	Ectonucleotide pyrophosphatase/ phosphodiesterase 2	1.00	0.33	0.20	0.21	0.31	0.24	0.19
**NEFL**	Neurofilament, light polypeptide	0.03	1.03	0.06	0.05	0.13	0.04	0.06
**OMD**	Osteomodulin	0.27	0.04	0.09	0.03	0.28	0.04	0.11
**ASPN**	Asporin	0.05	0.07	0.15	0.25	0.16	0.03	0.02

Table S8: Functional cluster analysis of ATRA regulated genes in WT cells

	Functional cluster	Enrichment score
> 2-fold regulated	Cell cycle, mitosis	19.4
	Chromosome segregation	8.9
	Extracellular matrix	8.5
	Angiogenesis	6.2
	Cytoskeleton organization	4.7
	Cell adhesion	4.3
	Neuron differentiation	3.3
	Response to steroid hormone stimulus	3.1
	Mesenchymal, neural crest cell differentiation	2.8
	Neuron projection	2.6
	Regulation of ossification	2.2
	Regulation of apoptosis	2.2
	Kidney development	2.0
	Response to vitamin A	1.9
	Retinoid binding, metabolism	1.5
> 4-fold regulated	Extracellular region	5.4
	Regulation of ossification	2.4
	Cell adhesion	2.1
	Mesenchymal, neural crest cell differentiation	2.0
	Muscle cell differentiation	2.0
	Blood circulation	1.7
	Retinoid binding, metabolism	1.5
	Neuron projection	1.5

Table S9: Real-time RT-PCR primers and conditions

Gene	Primer name	Primer sequence	PCR conditions
ALDH1A1	hALDH1A1-real1<br>hALDH1A1-real2	CGTGGGGGAATAAAGGCTAC<br>CATGATTTGCTGCACTGGTC	2step, 60°C,<br>Ethylenglycol
ASPN	hASPN-real-5'<br>hASPN-real-3'	TCCAACAAGAGAGCCAAGAAG<br>AATGTTGGTTGGGACTGAGG	2step, 60°C
CLSTN2	hCLSTN2-real-5'<br>hCLSTN2-real-3'	GACCAAGTCTCAGATAAGGAGCA<br>GCACACGGAGATGATGATGA	2step, 60°C
CPZ	hCPZ-real-5'<br>hCPZ-real-3'	ATTCGGCAAGCCAGTCAA<br>GGGCAGCAGTCTCCAGTAGT	2step, 60°C
CRABP2	hCRABP2_5'real<br>hCRABP2_3'real	CCAACTTCTCTGGCAACTGG<br>TCCTCAGCATCACATTCACC	2step, 60°C
CTGF	hCTGF-real1<br>hCTGF-real2	GAAGAGAACATTAAGAAGGGCAAA<br>ATGTCTTCATGCTGGTGCAG	2step, 60°C
DKK1	hDkk1_3'real<br>hDkk1_5'real	ACCCATCCAAGGTGCTATGA<br>GGGAATTACTGCAAAAATGGAA	2step, 60°C
DPT	hDPT_5'real<br>hDPT_3'real	CGAGGAGCAACAACCACTTT<br>CATATGTGGACACCCTCCTGT	2step, 60°C,<br>Ethylenglycol
ENPP2	hENPP2-real1<br>hENPP2-real2	TCCTAATGCCACTCTGGACA<br>ACTCGGAGAAACACGGACAT	2step, 60°C
EPHA4	hEPHA4-real-5'<br>hEPHA4-real-3'	GACAAACTCATCCGCAACC<br>TCCATTTTAATGGCCTGGAG	2step, 60°C
EZH2	hEZH2-ex7-real5'<br>hEZH2-ex8-real3'	GCCATTTCCTCAATGTTTCC<br>TCCATCTATGTTGGGGGTACA	3step, 58°C 10sec,<br>72°C 10sec
HPRT	hHPRT1<br>hHPRT2	TGACACTGGCAAAACAATGCA<br>GGTCCTTTTCACCAGCAAGCT	2step, 60°C
IGFBP3	hIGFBP3-real1<br>hIGFBP3-real2	GGGGTGTACACATTCCCAAC<br>AGGCTGCCCATACTTATCCA	2step, 60°C
MAD2L1	hMAD2L1-real1<br>hMAD2L1-real2	TGTTGGAAGTTTCTTGTTCATTTG<br>AAGGCGGACTTCCTCAGAAT	2step, 60°C
MGP	hMGP-real-5'<br>hMPG-real-3'	CCTTCATATCCCCTCAGCAG<br>GCGTTCGCAAAGTCTGTAGTC	2step, 60°C
NEFL	NEFL-RT-PCR-l<br>NEFL-RT-PCR-r	CATCAGCGCTATGCAGGAC<br>TAGGCAGATCGGCCAAAG	2step, 60°C
MYCN	hMYCN-real1<br>hMYCN-real2	CACAAGGCCCTCAGTACCTC<br>TTCTCCACAGTGACCACGTC	2step, 60°C
NRG1	hNRG1-real_F<br>hNRG1-real_R	TCAGTATCCACAGAAGGAGCAA<br>ACTCCCCTCCATTCACACAG	2step, 60°C
OMD	hOMD-real-5'<br>hOMD-real-3'	CACAACAAATTGAAGCAAGCA<br>TTTGGTCCACACGAATGTATGT	2step, 60°C
PLN	hPLN-real-5'<br>hPLN-real-3'	CCCAGCTAAACACCCGTAAG<br>AGCTGGCAGCCAAATATGAG	2step, 60°C
PRAME	hPRAME-real1<br>hPRAME-real2	CGTGGCAACAAGTGACTGAG<br>TATCGGCTCTGAATGGAACC	3step, 64°C 15sec,<br>72°C 15sec
RAMP	hRAMP-real1<br>hRAMP-real2	TGGGCCTCTCAGAAGAAAAA<br>GGGGAAGAATTGGATGGATT	2step, 60°C
RARA	hRARA-real1<br>hRARA-real2	GACCAGATCACCCTCCTCAA<br>GTCCGAGAAGGTCATGGTGT	2step, 60°C
RARB	hRARB-real1<br>hRARB-real2	CAAGCCTCACATGTTTCCAA<br>ATGAGAGGTGGCATTGATCC	2step, 60°C
RARG	hRARG-real1<br>hRARG-real2	CAGCCCTACATGTTCCCAAG<br>GCCTGGAATCTCCATCTTCA	2step, 60°C
RARRES1	hRARRES1-real1<br>hRARRES1-real2	CAACTGAAAAACCCCTTGGA<br>GAAAGCCAAATCCCAGATGA	2step, 60°C

RARRES2	hRARRES2-real1<br>hRARRES2-real2	GAAGAAACCCGAGTGCAAAG<br>TTTGTCCTCAGAGCCCAGTT	2step, 60°C
RARRES3	hRARRES3-real1<br>hRARRES3-real2	TGTGAGCAGGAACTGTGAGC<br>GCCACACCAACTTCAACCTT	2step, 60°C
SFRP4	hSFRP4-real1<br>hSFRP4-real2	CGCTCAAGGATGATGCTTCT<br>GAACTGTTCTCCGCTGTTCC	2step, 60°C
SRGN	hSRGN-real-5'<br>hSRGN-real-3'	GCCTTGAAGAAAAAGGACCA<br>CCGAAGCCTGATCCAGAGTA	2step, 60°C
SULF1	hSULF1-real-5'<br>hSULF1-real-3'	TAAAGATGGAGGAAGCTATGACC<br>CTCTAGGCCTTGCCAGTTGA	2step, 60°C
TGM2	hTGM2-real-5'<br>hTGM2-real-3'	GCTACCAGGGATCCAGCTTT<br>ATACTCCTGCCGCTCCTCTT	2step, 60°C
THRA	hTHRA-real1<br>hTHRA-real2	TCGAGCACTACGTCAACCAC<br>CCCCCTTGTACAGAATCGAA	2step, 60°C
TNC	hTNC-real-5'<br>hTNC-real-3'	CCGGTTACCTGCTGGTCTAT<br>CCAGGCTGTAGGAGGTGGTA	2step, 60°C
WNT2B	hWNT2B-real-5'<br>hWNT2B-real-3'	CACCCGGACTGATCTTGTCT<br>GCCACAGCACATGATTTCACA	2step, 60°C
