# Supplementary material for: Retinoic acid pathway activity in wilms tumors and characterization of biological responses in vitro
Source: Mol Cancer. 2011 Nov 8;10:136. doi: 10.1186/1476-4598-10-136 (PMC3239322; doi:10.1186/1476-4598-10-136)
Supplement: Additional file 2 — Figure S1. All cell cultures derived from tumors of one patient reacted in the same way to RA treatment. Ws568reA and ws568reB showed reduction of growth rate under RA treatment like ws568li did. Figure S2. Low ATRA concentrations are sufficient to reduce cell growth. ws568li cells were treated with different ATRA concentrations, which all reduced cell proliferation to the same extent. Figure S3. SA-β-Gal staining of RA-treated ws568li cells. ATRA and 9cisRA induced only few senescent cells after 4d of administration while 4HPR treated cells showed no senescence. Figure S4. Caspase3 IHC staining of ws539A cells after 4d of RA treatment. Fenretinide and the combination of 4HPR and the HDAC inhibitor SAHA induced apoptosis in primary WT cells while ATRA, 9cisRA and ATRA combined with HDAC inhibitor did not. [file 1476-4598-10-136-S2.PDF]

Additional file 2

Fig. S1

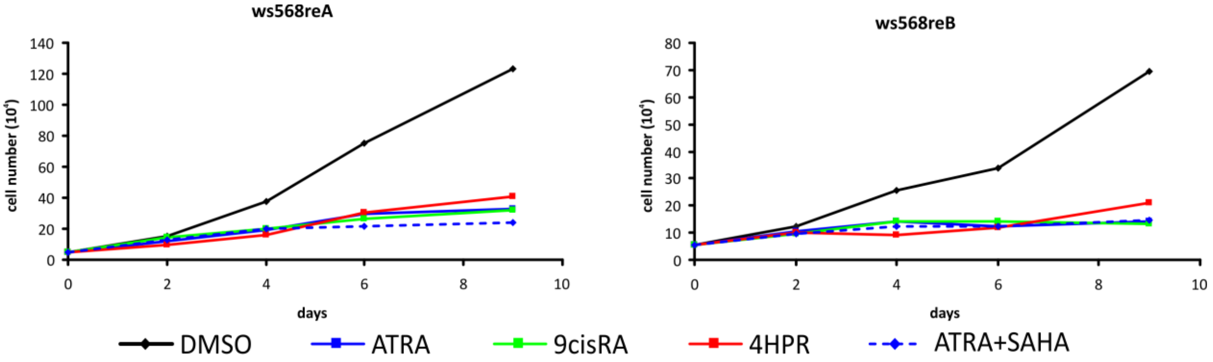

All cell cultures derived from tumors of one patient reacted in the same way. Ws568reA and ws568reB showed reduction of growth rate under RA treatment like ws568li did.

Fig. S2

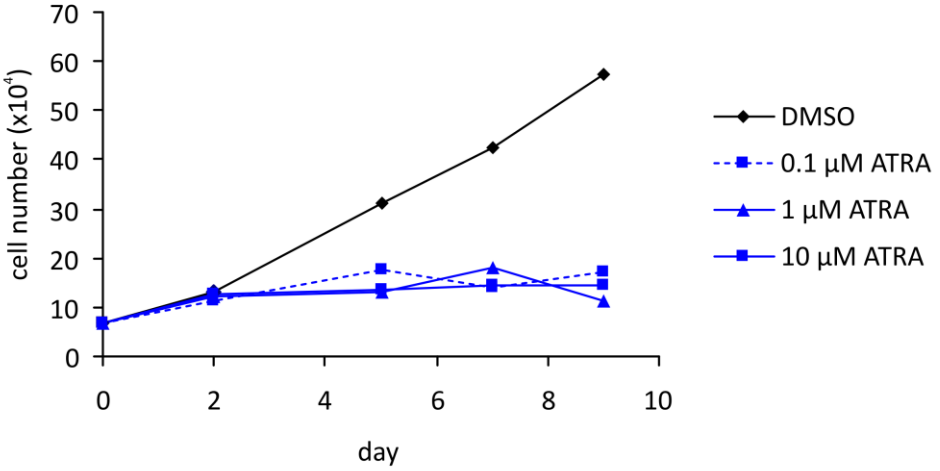

Low ATRA concentrations are sufficient to reduce cell growth: ws568li cells were treated with different ATRA concentrations which all reduced cell proliferation to the same extent.

Fig. S3

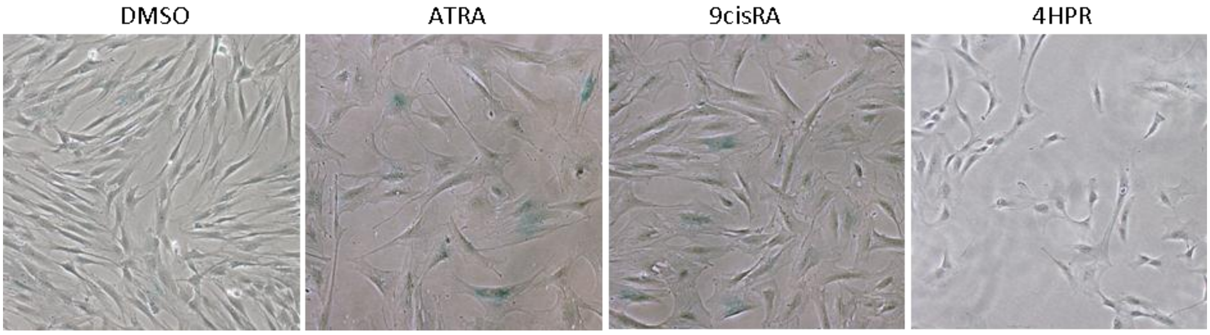

SA-β-Gal staining of RA-treated ws568li cells. ATRA and 9cisRA induced only few senescent cells after 4d of administration while 4HPR treated cells showed no senescence.

Fig.S4

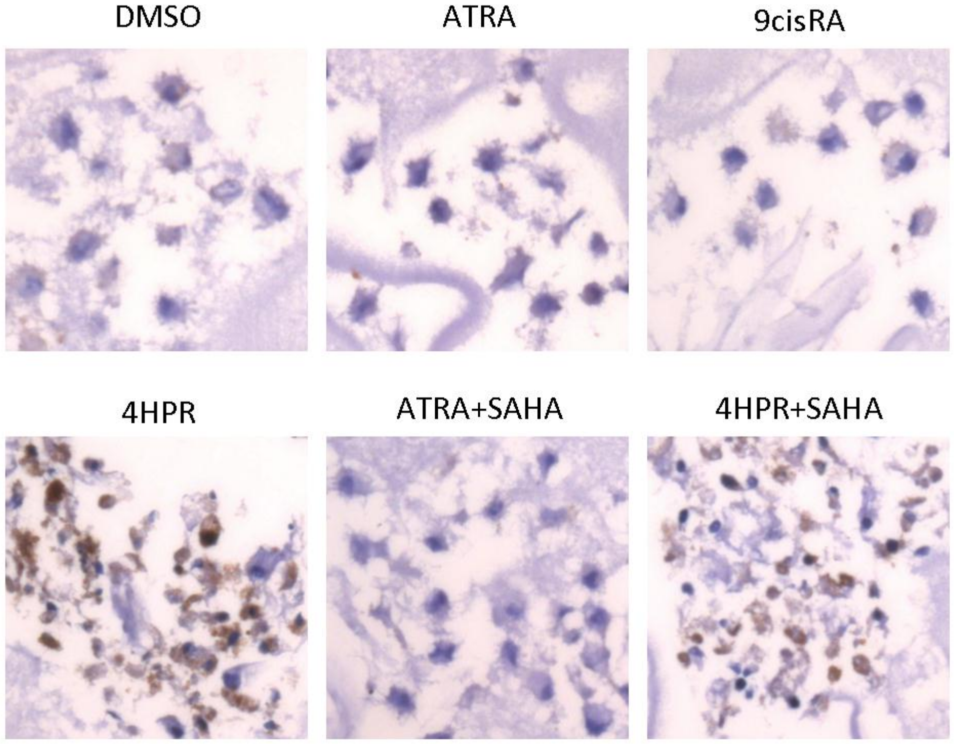

Caspase3 IHC staining of ws539A cells after 4d of RA treatment. Fenretinide and the combination of 4HPR and the HDAC inhibitor SAHA induced apoptosis in primary WT cells while ATRA, 9cisRA and ATRA combined with HDAC inhibitor did not.
